# Tropical Diseases Met with in England

**Published:** 1909-11-27

**Authors:** James Cantlie


### Hospital Clinics.

### TROPICAL DISEASES MET WITH IN ENGLAND.

BY JAMES CANTLIE, F.R.C.S.Eng., etc.

(A Lecture delivered at the Medical Graduates' College and Polyclinic.)

It does not matter what part of this country you may be practising in, you will be faced by a man who has come from abroad and develops illness or fever of some kind. You will find the patient seems very learned with regard to his own troubles; people who are home from the East can tell the doctor all about tropical disease, and the doctor has a troublesome time with them. Probably your patient has something in his face which stamps him as having been in the Tropics. Is it disease, or is it sun? It is not the sun, but it is something which disease has placed there, and which, for lack of a better name, I call the "Tropical Mask." It is usually associated with a form of anæmia in which the blood corpuscles have lost their hæmoglobin; it is that hæmoglobin deposited in the skin which forms the tropical mask. You ask where he has been, and remember the two great classes of diseases which you have to bear in mind are some form of fever, of which we will say malaria is the type, and secondly, some intestinal trouble, which we will call dysentery. These constitute the sum and substance of tropical diseases when you come to treat them practically. Enteric he may have had, but that does not cause lasting trouble.

First ask whether he has had malaria; 60 per cent. of people say no, yet they may be full of malaria parasites. Then ask as to dysentery. When he says he has not had malaria you have to try and prove to him that he has. He may say he has had fever, and you are inclined to attribute everything to malaria. What does he mean when he says he has not had malaria and yet has been invalided home for fever? He will say he has had West Coast fever, or Singapore fever, or Shanghai fever, or, if he has been in Guiana, he will say he has had "the local fever." It is the way with people here at home. Ask them if they have ever had indigestion, and they will indignantly say "never!" yet you will find many of their teeth decayed and their stomach dilated, and they never knew what it was to digest a meal properly in their lives. People who say they have not had malaria, but local fever, have had malaria. Treat him for malaria, no matter what he says. Your next step is to take his temperature. It may be normal, or it may be above or below normal. Then you examine him. Watch him take off his clothes, and you will see he has on less clothes than the ordinary man. He has on no drawers and no vest, and perhaps only a thin suit; whereas this weather most men wear drawers and a fairly thick vest. One of the most common causes of relapses of fever in this country is insufficiency of clothing. Why is that? They do not feel cold. Ask such a patient how he came to you, perhaps on a cold day. He will reply on the top of a bus, and that very fact shows he probably has a touch of fever. They have such a store of heat in the body that they do not feel the cold like others do. Tell such a man that the sooner he takes to drawers and pants the better, or he will get frequent relapses of fever. Everyone who comes home from the tropics may have malaria in the blood, and if a chill or anything upsets him he may have fever. A man may never have had an attack of ague or fever, but yet on coming to England may develop one for the first time. It is quite common for people coming home from the tropics to have their first attack of fever at Marseilles. An enterprising doctor could set up a hospital there for such people and have a good practice. I knew a well-known man who lived in India all his days, and never had fever until he was coming up the Channel, and then he had a filarial attack of fever, and there was in him an impending elephantiasis of his scrotum.

When the man has taken off his clothes, you set to work to examine his abdomen. You need not bother about his heart; he was probably examined before he went out, and there are no athletics likely to be carried out in the tropics which are likely to damage his heart; there is practically no rheumatic fever; that diminishes the nearer you approach the tropics. Look to his liver and spleen, and always percuss the stomach and try to elicit the splash. The spleen may be enlarged, just below the costal cartilages. Feel well forward for an enlarged spleen, because it does not go downwards at first, but advances forwards on to the stomach. It is because the spleen is supported by the costo-colic fold, which supports it as in a cup. It cannot enlarge upwards because of the diaphragm, nor downwards because of the costo-colic fold; it can only go forwards. Having enlarged to the edge of the costo-colic fold, it falls down towards the iliac fossa. If it is very large it may be beyond the umbilicus. The spleen may be tender, but more often it is not; in chronic malaria the spleen need not be tender. There may be passive enlargement of the spleen. Next, feel for the liver. There may be no tenderness of it in malaria, and it may not be enlarged. You cannot rely on the condition of any other abdominal organs to tell you that a man may be suffering from chronic malaria. In chronic malaria the spleen is always enlarged.

Examine the heart, lungs, urine, and blood, and let the blood be examined by someone who knows the malaria parasite when he sees it. There are not many men whom you can trust to give you an exact report of the malaria parasite; I do not undertake to examine the blood, although I know the parasite. One should always take a specimen and examine it oneself first; but I always send my specimens to a better man for his opinion. If the patient is a fidgety man, what will you do until you get the report of the blood examination? If he is sensible he will wait until you get that report. If he is not sensible there can be no harm in giving him quinine; but only give it in a tentative way until you are sure what it is he has got. Should you give a man who has not had an attack for weeks quinine straight off? And where should he live? If he has not had fever for a fortnight or three weeks, which often he has not, a safe thing to give him is quinine twice a week. Quinine lasts about four days in our blood, so to keep it in a state of constant saturation give him quinine every fourth day. In that way you can anticipate the presence of malaria parasites set free in the blood. If you find he gets an attack of fever, what then? He should go to bed, and then take his quinine every day for three days, and three times every day; seven grains each time. The quinine is better given in a mixture, not in tablets or tabloids, nor even in a powder; it is much more efficacious in acid and water. If there is so much sickness as to interfere with the administration of the quinine, inject intramuscularly the bi-hydrochloride of quinine: but as a rule patients will keep quinine down quite well.

You have the case of a patient who has been home four months, and his tropical mask remains; he has not had shivering, and he is not ill enough to be properly under your care, and yet he is not well enough to go about without any attention at all. He is allowed to look after himself, which generally he does not; and it is perplexing to know what to do. In chronic malaria what should one do? Give quinine, as I say. Is it of any use to give anything else? We used to give opium, always anticipated by calomel. In bad malarial attacks and relapsing fever it is a good plan to commence with 5 gr. doses of calomel, followed four or five hours afterwards by Epsom salts. That cleans the tongue, but the solution of mercury in the blood lasts three days. Quinine is another strong drug which acts on these parasites. You find it has been the custom to give morphia and opium, and then you should give the quinine. Some even add arsenic in small quantities. These together should make an excellent pill; and if you find frequent relapses of malaria, that pill should be given three times a day.

There is another form of fever. A man comes home with a big spleen, and you ask what you shall do with him. Drugs will not do very much, except to counteract his attacks of fever. Will you send him to the seaside? That is the worst place. Many people from the tropics stop at the Riviera, perhaps to cool down; those who have done it do not repeat it, but they regret it. A man who has a big spleen wants snow and ice, a climate which is the antithesis of that which he came from. A man with chronic malaria and a big spleen gets very ill, but on a frosty day he is magnificent. The first time I came home I had a big liver and spleen. On a warm day I went to Henley, and I came back with fever which lasted me for two months; I had a temperature every day for two months. You will send such a man to the Yorkshire moors, or to Switzerland in January and February. Let him go tobogganning and join in the sports going on there. The way in which such a man gets rid of his trouble there is wonderful. Four or five years ago a man from the West Coast of Africa came to me with a big spleen, and a history of constant relapses of fever. Never let such a patient stay in town, especially in the summer time, but send him up to the heights and the bracing places. I asked this man where he was going, and he said "To Switzerland, whatever you say." I told him I was longing for him to go. I had not yet prescribed Switzerland for anyone with a big spleen. When he had been out there a week or two he was so much better that he wrote to a friend who had also returned from the tropics, and asked him to come, and when they returned, two healthier men could not be found. They went out to the West Coast again, and kept quite well. The patients having found their way to Switzerland when they have chronic malaria, we send them ourselves.

But if a man has a tropical mask and a big spleen and yet he has only three months' leave, is he fit to go back? No. You keep him at home until his spleen has gone down and he can take walks without a sense of fatigue, and that matter of fatigue is the best test. The first summer I came home, I tried to shoot, but an hour and a half was enough. The second summer I could manage three hours, and the fourth summer I could go on from eight in the morning until eight at night. But necessity generally drives, and you can only advise your patient to the best of your ability according to his leave. He must be advised to protect himself with quinine, which should be renewed every fourth day. By this time you will have got your report as to whether it is malarial parasite or not, and if it is you can give him confident directions. May this condition be anything but malaria? Yes, it may be Malta fever. A doctor in Bucks had a man come to him from the Riviera, with febrile trouble. His temperature was taken, and it showed a paroxysm of fever every day. Specimens of his blood were sent up for examination, and it was not malaria. He had been given seven grains of quinine three times a day for three days, and there was no marked effect in the temperature, and when you find that is so, the condition is not malaria. Then the doctor thought it was enteric, and sent some blood to be tested for that; but it was not that. Then he sent more blood for examination and asked whether it was Malta fever. That is what it was pronounced to be. He was quite right in his steps: first malaria, second enteric, third Malta fever. You should specially think of Malta fever if the man has come from the shores of the Mediterranean, or if he comes from Ceylon. Quite recently I saw a man from Somaliland who had Malta fever, for they have it there. You can only confirm it by blood examination. The undulant temperature will tell you largely. What will you do for Malta fever? I hope you have not had such a case to treat, and that you will not do so. If you do, it will often be a case which someone has had to treat and got tired of; it is so prolonged and so annoying, because there is so little that can be done for it. Injections are used for it, but I have not seen much good from them. You have to try to gain the confidence of the patient. You may be seeing him every day. Tell him the meaning of Malta fever, and interest him in his disease; tell him the difference between a vaccine and a serum, and do your best for him. Otherwise you may lose him. One man in the country was being treated by his local doctor, and a London man was called in, and he told him he must come up to town to be treated. I was called in, and advised the patient to go back to the country. In Malta fever you must treat the symptoms as they arise; and there are generally plenty of them.

But you may find from examination of the blood that it is not Malta fever. If it is not, you fall back on the condition of the liver: it may be a liver abscess all the time. Quinine has no effect upon it, and the blood shows no sign of enteric. You find a tremendous amount of sweating, more than in Malta fever, and even more than in malarial fever. In liver abscess there are early morning sweats and wasting, and there may be some local pain. There need be no enlargement of the liver below the costal cartilages. There may be congestion at the base of the lung, which is on account of the liver condition. This lung congestion is one of the troubles in tropical diseases. When the liver is congested, it is heavier, and then it is more difficult for the liver to move with the diaphragm on that side, and that muscle becomes exhausted. If there is the least bit of pain, the diaphragm will not enable you to take a deep breath. When the diaphragm refuses to act, the liver will recede beneath the diaphragm, and with a couple of pints of pus in the diaphragm, the liver will be small. If the lung is not moving, it will be congested. So a man with night sweats, and with a daily-recurring fever and the other conditions I have mentioned, has a liver abscess. What will you do for him? Put a needle in as soon as you can. When you are searching for pus you must be prepared to operate straightaway. We have not time to go into that to-day. When you have eliminated other things down to this, you get someone to help you. If you suspect abscess you must not needle to-day and have a surgeon down to-morrow, because by then pus may have extravasated into the peritoneum and then you will be too late. None of those three conditions can be diagnosed straightaway. If a patient says he has had fever every second day, you do not need to be told what he has got.

I have not time to go into such things as filaria, but that is so unlikely that it need hardly be taken into consideration. He may have chyluria, and then you suspect filarial poisoning, and you can easily diagnose it. On examining the blood after 12 o'clock at night by the microscope, you can easily see this embryo worm. Filarial fever may come on at irregular intervals; it is very high, and nothing is effectual in it. There is great pain in the loins, and difficulty in passing water, and when passed it contains chyle. The first time I examined a case I found many small worms in the urine, and I had never heard of such a condition, and so thought I had discovered a new disease. Sir Patrick Manson came in, and quickly put me right.

Now about dysenteric troubles. A man tells you he has been invalided home with dysentery; you had better see his stool. But he may be staying in the country, and may be going back in an hour or two, so you do not see his stool. He says he does not feel very bad, but he may be prolonging his disease. You tell him that you must see him again, as it is hopeless unless you see his stool. He says the first stool is loose, but the next is well formed. His bowel is not right. If he is really ill you get him to bed, and then you can treat him; but with such a trouble which is not acute you are in a difficulty. Mere blood and mucus will not diagnose the condition, but the blood must be incorporated with the mucus. If there is merely blood on the mucus, the condition may be ordinary colitis. The best definition of a healthy stool which I can give you is that it is one which sinks in the water, and is round and well-formed. If it floats it is not a healthy one, and if it has fermentation in it, it will not sink. This man's stool does not sink in water, and you put him to bed immediately. He may develop acute dysentery at any moment. The next step is, give him ipecacuanha at once; that is good enough to go on with. It seems to attack the amœba of dysentery; at any rate, the point is it does good. Give the powdered root in doses of 30grs. If it makes him sick, that probably is not harmful. Our forefathers first gave the patient an emetic and then bled him. The ipecacuanha should be kept down for some time: if not give ipecacuanha without emetine; only one or two chemists in London seem to keep it, and none in the country. Give 20ᵐ laudanum first at, say, 20 minutes to 12; at 10 minutes to, give some ice to suck; then put mustard on to the stomach, and at 12 o'clock give 30 grs. ipecacuanha without emetine. Give some food a short time afterwards. Do not keep these patients starving too long. You are always safe to give some calf's foot jelly, which leaves no residue. Milk has a residue, so has beef-tea. Next day give 30 grains of the drug, next day reduce to 20 grains, next day to 15, next day to 10. Keep the patient on 10 grains for a week or more. You need observe no precautions in regard to laudanum. Sometimes ipecacuanha produces diarrhœa; a thin stool, which is fæcal but fluid, and dark from the presence of bile; no shreds, no mucus, no blood.

If the patient has not got shreds or mucus, but has alternating constipation and diarrhœa, you think of worms. But you will seldom hear people admit they have had worms: they deny worms almost as they deny drink. One woman was sent from China to Japan for change of air—" change of water " the Chinaman calls it, and he is often right—and then was sent to England, because of her dysentery. On the way home she vomited a worm, and she arrived in England quite well. A dose of santonin when she was at home would have saved all that trouble. Some of these people have bottlefuls of worms in their intestines. One Chinese boy at ten had worms, and when I went to see him he had one and a half bottlefuls of them for me to look at. I could not tell you how many there were. In his case I stopped the santonin, because I believed they caused the worms. We were in a district where worms and santonin were as common as peas, and yet we took the woman's case to be dysentery when it was really worms. So when you get a patient with intestinal flux which you cannot make head or tail of, give santonin. Give six 3-gr. doses of santonin. Give santonin night and morning, and on the third morning give castor oil; *i.e.* three doses of santonin, one dose of oil; three more of santonin, one of oil. You may say you can examine the fæces for eggs. So you can, but it is easy to make mistakes as to what is and what is not an egg. Do not rely upon your microscope, but get expert opinion. Even three experts may not agree. One will be sure it is vegetable, the second thinks it is an egg, and the third will not be sure what it is.

You find a patient who has diarrhœa one day, and constipation another, and that he is passing blood and mucus, or it may be, blood and no mucus. His trouble is five or six inches up from the rectum. Put your finger into the rectum, and what do you feel there? If you were blind-folded and were asked to pass your finger up into such a condition you would say you were feeling a vagina and uterus, with a protruding os. That was the condition we found in a man who was passing stools occasionally; irregular stools, sometimes fairly formed, and soft. But when he had finished there was mucus and some blood on top of it. It is a common occurrence in the post-dysenteric state. Dysentery commences at the anus, and travels upwards. In malignant dysentery the intestine gets black and gangrenous up to the commencement of the small intestine, and then the patient dies. But in chronic dysentery, in which the ulcers have left cicatrices, the fæces formed above push the narrow bowel into the upper part of the rectum and invaginate it. What can be done with it? If you pass a tube up there, you do great good by reducing the intussusception, but in 99 times out of 100 you will get nothing through. You wash it out frequently by injections from below. The best thing to use is sea water: not Tidman's salt added to water; there is some beneficial property in sea water which we cannot imitate, and you can get sea water delivered at your door for a few pence per gallon. But do not keep it longer than a week, as it decomposes. The bleeding ceases almost immediately after you begin to use it, and the ulcerated condition subsides to it better than to anything else. The diet does not matter very much. The patient may be looking much better, but still there may be the passage of mucus and blood every day. Always begin with santonin, then give ipecacuanha, then wash out the bowel, and feed the patient well with vegetables and fruit, especially if they are somewhat scorbutic. This country does them good, because they can get plenty of fresh fruit and vegetables. Then in regard to lactobacillin. I introduced that into this country, and I thought it was the key to dysenteric troubles; but it is very disappointing.

Sprue I have not time to speak of. I have asked a well-known chemist to give me a milk with an alkaline fermentation in it. Sprue always does harm in acid fermentation. You have a man æt. 55 who is played out, and who has loose stools because he cannot digest his food. His skin is parched and dry, and he is looking worn out. On him lactobacilline acts like a charm; but for acid diarrhœa and sprue you will find acid fermentations worse than useless.

